# Public and patient involvement: exploring public partnership in pathogen whole-genome sequencing research and its data visualisation

**DOI:** 10.1099/mgen.0.001691

**Published:** 2026-04-09

**Authors:** Suzanne Rotheram, Hannah Trivett, Jane Whitehurst, Caitlin Collins, Jake Carson, Sophie Staniszewska, Xavier Didelot

**Affiliations:** 1 Department of Public Health, Policy and Systems, The University of Liverpool, Liverpool, UK; 2 Department of Microbes, Infection & Microbiomes, School of Infection, Inflammation and Immunology, College of Medicine & Health, University of Birmingham, Birmingham, UK; 3 Public Contributor, Warwick, UK; 4 UK Health Security Agency, London, UK; 5 Mathematics Institute, University of Warwick, Coventry, UK; 6 Warwick Medical School, Division of Health Sciences, University of Warwick, Coventry, UK; 7 School of Life Sciences and Department of Statistics, University of Warwick, Coventry, UK

**Keywords:** data visualisation, pathogen genomics, patient and public involvement (PPI), whole-genome sequencing (WGS)

## Abstract

Pathogen genomics is increasingly used in publicly funded studies and has important implications for understanding infectious diseases and their spread. However, unlike many other research areas, it has seen little patient and public involvement (PPI), thereby missing opportunities to enhance both research processes and outcomes. This project addressed that gap by exploring the potential contribution of PPI to pathogen genomics, using whole-genome sequencing (WGS) data visualisation as an exemplar.

Following ethical approvals, three 2 h PPI workshops involving five public contributors and six academic contributors were held online. Sessions were documented using visual meeting notes. Workshops were audio-recorded, transcribed and analysed using an iterative thematic analysis.

Two interconnected themes were identified. First, effective public involvement required collaborative sense-making, achieved through co-producing a shared knowledge base, establishing consistent terminology and developing effective practices for knowledge exchange. Second, participants highlighted three priority areas for meaningful PPI in future pathogen genomics research: (i) prioritising research questions, (ii) contributing to decisions about data collection and use and (iii) supporting the communication of findings.

Although pathogen genomics is technically complex, this did not prevent productive discussion about how, where and why PPI could be integrated into research. Expanding PPI in this area could help align pathogen WGS research with patient priorities, inform approaches to data governance and improve the accessibility of research outputs to the public. Realising this potential, however, will require active engagement from researchers in the field.

**Impact Statement**

This is the first study, to our knowledge, to identify when, where and how patient and public involvement (PPI) could be involved in microbial genomics research. We show that meaningful PPI is feasible even within technically complex areas such as whole-genome sequencing data interpretation. Through co-produced workshops, we identify three key opportunities for PPI in pathogen genomics: elaborating on research questions, informing decisions about data collection and governance and improving the communication of results. These findings provide an exemplar of how public perspectives can be integrated into discussions around microbial genomics and show ways in which PPI could enhance the relevance, transparency and accessibility of pathogen genomics research.

## Data Summary

The anonymised data supporting this study are openly available from the University of Liverpool Research Data Catalogue at https://doi.org/10.17638/datacat.liverpool.ac.uk/3075.

## Introduction

Patient and public involvement (PPI) in research is defined as ‘an active partnership between patients and the public and researchers in the research process’ [1,2]. PPI is not research that involves members of the public as study participants [3]. It is also distinct from activities that emphasise sharing research findings with the public, often referred to as public engagement [4].

The last 20 years have seen a marked expansion of PPI within research activities, and PPI is now often a requirement of research councils because of the many positive impacts it can bring [5,6]. For example, public involvement can help to hone research questions and outcomes, ensuring that research is relevant to the people who will be affected by it or use it [7,8]. PPI can improve the research experience for study participants, bring insights into what research is acceptable to the public and improve recruitment and retention in studies [9]. Members of the public can also help researchers improve the way research findings are communicated and used [5,8, 9].

PPI is already effectively integrated into many areas of research, particularly clinical, health and social care [5,9, 10]. There is a mixed and limited picture, however, with respect to PPI in genomics research. While PPI is regularly integrated into human genomics research [11,14], there appears to be a notable gap in PPI within pathogen genomics. A literature search yielded limited evidence of patient or public involvement in pathogen genomics research. Only one relevant commentary was identified, referring to public involvement in the sequencing of *Clostridium difficile*; however, it did not clearly describe how the public contributed to the research process or demonstrate meaningful involvement [15]. Public involvement is also notably absent from pathogen genomics policy documents, such as the World Health Organisation (WHO) global genomics surveillance strategy for pathogens with pandemic and epidemic potential, and the current UK Health Security Agency (UKHSA) pathogen genomics strategy [16,17].

The relative absence of PPI in pathogen genomics is concerning, considering the rapid and valuable growth of this research area in clinical diagnostic work and routine public health practice, especially following the coronavirus disease (COVID-19) pandemic [18,19]. Without active efforts to involve the public, pathogen genomic research risks missing the benefits that public involvement has brought to other research areas [5].

This project aims to address the gap in PPI in pathogen genomics by focusing on pathogen whole-genome sequencing (WGS), which is now well-established and conceptually simpler than previous molecular typing methods. As minimal PPI research has been published within this area, this project focuses on WGS data visualisation to provide a more accessible starting point for discussions within the scope of this project. Our aim was to ‘explore and capture the potential contribution of patients and the public to pathogen WGS and use pathogen WGS data visualisation as an exemplar for future public involvement work’.

## The project team and PPI meetings

The project team comprised five public contributors and six researchers (‘public’ and ‘academic’ contributors, respectively) [20]. Public contributors were recruited through the National Institute for Health and Care Research (NIHR) Health Protection Research Unit in Gastrointestinal Infections PPI Group [21], contributing lived experience and a wide societal perspective. One public contributor (J.W.) was involved throughout the project, including planning and manuscript preparation, while the remaining four attended workshops. Academic contributors brought expertise in PPI, qualitative research and pathogen genomics. S.R. facilitated the sessions, S.S. advised on PPI development and X.D., H.T., J.C. and C.C. attended two to three workshops each, so there was an equal balance of public and academic contributors in each session.

PPI meetings were held on three occasions over 9 weeks, each consisting of a 2 h online Zoom session. Before each meeting, slides and a content overview, developed by academic contributors and J.W., were circulated to attendees. A visual artist recorded ‘visual minutes’ of each session (Appendices A–C, available in the online Supplementary Material), and a ‘living dictionary’ was developed throughout the discussions to explain key terms (Appendix D).

Meeting one started with a discussion about the basic science behind pathogen genomics, the motivations behind conducting pathogen WGS analyses and the different stages of applying pathogen WGS in a laboratory setting (Appendix A). Meeting two covered the applications of pathogen WGS and the use of phylogenetic trees (Appendix B). Meeting three started with more discussions and examples of phylogenetic trees. It finished with a discussion of the NIHR PPI research cycle to identify possible areas of public contribution in pathogen WGS research [22] (Appendix E).

Each meeting was recorded, transcribed and analysed using an iterative thematic analysis [23]. The analysis identified two main interconnected themes. First, effective public involvement required collaborative sense-making. Second, participants highlighted three priority areas for meaningful PPI in future pathogen genomics research.

It is important to note that this work was not a quantitative study, but a PPI project. Its purpose was to inform and refine future research practice, rather than to generate primary data, produce generalisable findings or meet the methodological standards expected of hypothesis-driven research. The themes presented below should therefore be understood as insights arising from an involvement activity, rather than as findings from quantitative research. Given the small number of public contributors, we are unable to make claims on bias or representativeness.

## Engaging in collaborative sense-making

The first theme identified how, before discussing public involvement in pathogen WGS research, public and academic contributors needed to collaboratively develop a shared understanding of how pathogen WGS worked in practice. This took time but was a necessary precursor to conversations about where or how PPI could be integrated into pathogen genomics. Across three sessions, this sense-making involved co-producing knowledge, challenging inconsistent language and identifying effective knowledge-sharing practices.

## Co-producing a knowledge base

Public contributors began the project with varying levels of knowledge of pathogens and genomics, requiring time to establish a shared understanding. For example, one public contributor explained that even terminology integral to the term ‘pathogen genomics’ was not immediately understandable:

*I don’t really know what pathogen means. I don’t know why we use ‘pathogen’ as opposed to ‘microbe’. If you were to ask me, what’s a microbe? I would say a tiny bacteria. I wouldn’t think of a microbe as being a virus, but then I wouldn’t have thought of pathogen as being a virus or bacteria either, so the words don’t really mean anything to me […] I don't have any sense for the history or why or wherefores*. (Public contributor 1)

In contrast, public contributor 2 found the explanations of pathogen, virus and bacteria easier to understand:

*I’m quite happy with the terminology*. (Public contributor 2)

As discussions progressed, the project team found that effective knowledge-building depended on academic contributors carefully pitching essential information. On the one hand, oversimplification could stop public contributors from contributing effectively. On the other hand, an ‘overload’ (public contributor 2) of difficult terms and concepts could cause confusion. For example, terminology used in a diagram of a bacterial plasmid presented by an academic contributor was difficult for public contributors to follow:

Public contributor 1: *Origin of replication, what does that mean?*

Academic contributor 5: *That’s just the start, that should just say, ‘start’. Again, it’s one of those situations where scientists like to overcomplicate it. You can just have that as – ‘this is the gene, that is the start of the sequence’*.

## Challenging inconsistencies in language

One cause of confusion in the workshops was inconsistencies in the language used in pathogen genomics. Academic contributors became aware of these inconsistencies when they were noticed and challenged by public contributors. For example, public contributor 1 pointed out that the word ‘base’, referring to a DNA base, meant something very different to the public. These different uses in different contexts could lead to confusion, as shown in this exchange about the terminology surrounding mutations:

Public contributor 1: *Does mutation always have a negative connotation? Because for me, mutation means bad, you know, you know, Ninja Mutant Turtles, or the mutants in X-Men, or what have you? […]*

Academic contributor 3: *So I would say in the context of bacteria mutation is definitely, not always a bad thing for them*.

The issue was exacerbated when academic contributors used interchangeable terms (for example, nucleotide/base/position/site), as in this example when discussing the use of the term ‘pathogen’:

*… we’ll start with pathogen. We’re using this term broadly and somewhat interchangeably with microbe*. (Academic contributor 3)

One public contributor suggested that it would be less confusing if a new vocabulary was developed for PPI work. There was an example here of how this might work in practice, referring to the terminology used in pathogen genomics of washing and elution in DNA extraction:

*I just think there’s a lot to be said for saying what you mean. So, we might need to recreate our own vocabulary, where, when you [say] ‘detach dirt’, you mean ‘detach dirt’. Not wash. […] Say what you mean and mean what you say rather than this historical vocabulary, which means so many things to different people*. (Public contributor 1)

## Finding effective ways to share knowledge

As the group worked together, it emerged that some practices were better at building a knowledge base than others. For example, when academic contributors tried to explain phylogenetic trees from first principles, public contributors were confused. When these same principles were explained using concrete examples, such as a real-life outbreak, there was a better understanding, leading to this reflection from one academic contributor:

*I think maybe what we’re learning is that maybe it’s best to look at a real example and explain that in detail rather than explain from basic principles. That’s something we take away today, maybe*. (Academic contributor 1)

Another method that helped bridge understanding between academic and public contributors was the use of analogies, which were used on multiple occasions. For phylogenies, comparison with family trees seemed especially useful. As another example, here, an academic contributor used a washing machine analogy to explain the steps needed to extract DNA:

*So, think of it like your washing machine with your detergent when you’ve got soil on your T-shirt, you want to break that apart so that it’s nice and clean. So, we’re trying to break apart. And then we’re trying to open it up so we can get the DNA out. And that’s what the detergent is, and it’s a similar concept of a detergent in an extraction*. (Academic contributor 5)

This use of the analogy then allowed a public contributor to build on the idea of a washing machine to help them gain a greater understanding:

*So going with your washing machine analogy. How do you know that the washing machine and the detergent and the heat and the water, and all the rest of it aren’t damaging the very thing that you’re trying to preserve?* (Public contributor 1)

Once a level of shared sense-making was established, public and academic contributors were able to explore the potential role of PPI in pathogen genomics. These discussions are presented below.

## Potential areas for useful public involvement

Using the NIHR PPI research cycle diagram (Appendix E), public contributors noted that PPI should be integrated across all pathogen genomics work. Three areas of PPI stood out and were explored in more depth. These were the prioritisation of research, the collection and use of data and research communication, described in more detail below.

## Prioritisation of research ideas

Involving the public in the preliminary stages of the research cycle was highlighted by one public contributor as being particularly important:

*…. the identification and prioritisation of projects for research is really, really important [for PPI work]*. (Public contributor 2)

Other public and academic contributors agreed that the public could be usefully involved in exploring in depth what the purpose of the research was at the start of projects, to make sure it had more impact:

Public contributor 1: *… maybe that’s the role of the public is to say, well, and so what? What are you going to do with this? You know why is it important? And try and work with you [academics] to develop that research question so that it actually does something. You know, not more meaningful, but […] to maximize the impact*.

Academic contributor 1: *That first step in the [NIHR] diagram of, establishing your priorities and deciding what is worth spending time doing, you know, clearly is super important and there’s a lot the public can help with on that*.

## The collection and use of data

One particularly fruitful area for discussion was the integration of PPI into making decisions about the use and collection of pathogen genomic data. Public and academic contributors came at these questions from different perspectives. Whereas academic contributors could be quite frustrated when they spoke about data, and how the type of data they had access to could limit their work, public contributors challenged this perception, suggesting that researchers, perhaps supported by the public, could play a more active role in the type of data they could access:

*I notice this a lot when I’m talking to researchers. They seem to feel that they are the recipients of data, and that they can only get the data that they can get […] and there never seems to be a two-way dialogue about well, actually, we’d be able to do better science with different types of data*. (Public contributor 1)

As this idea of researchers as ‘recipients’ of data was challenged, several opportunities were identified where public contributors could become involved. Public contributors said that the public would be able to work with researchers to decide what kind of information should be collected alongside samples and to think about how and where that data could be collected. Public and academic contributors recognised that there could also be a role for PPI work to question whose data are included and excluded in research studies, as well as decisions around how data are grouped into categories. These conversations then led on to ethical considerations in pathogen genomics; how pathogen genomic data are collected from individuals or groups, who has access to the use of pathogen genomics and how its use is communicated to the public. With respect to these considerations, public contributors had concerns:

*…. there’s one more [area of involvement] on the ethics level, how data is collected, how data is stored, what you do with it? We talked about access to treatment, who gets access to personalised medicine, who gets access to having their sample analysed? So, I think there’s an ethics branch of public involvement*. (Public contributor 1)

*… we need to be involved in the processes whereby that [the use of pathogen genomics in personalized medicine] is controlled as much as anything else. What the patient will learn and know from that information, and how it will be communicated to them*. (Public contributor 3)

Finally, public and academic contributors explored whether there was a role for PPI work in the building of genomic trees to add trust and validity to the models. Academic contributors explained that although the creation of the examples that were being explored in this project – phylogenetic trees – was technical, *‘at the level of the pathogen’* (academic contributor 1), there might be some role for the public in deciding what assumptions are used in a transmission tree:

*… that’s where I can see that, definitely, there would be some assumptions [about data] being made. […] And so, any input that the public can have on the validity of the models has an impact on how we interpret the genomic data. So yes, definitely, there is a scope for the public to be involved in that*. (Academic contributor 1)

## Communicating research

Academic and public contributors recognized that PPI could play a key role in communicating pathogen genomics research, including helping to prioritise which stories should be shared with the public or within research papers. PPI was also identified as supporting the communication of pathogen genomics research to the public in more accessible ways. For example, in relation to data visualisation, public contributors encouraged academic contributors to reflect more critically about how data were presented, and how accessibility could be improved:

*I think the diagram of the tree there is too complex. There’s too much information for you to take in. I think you could simplify it and perhaps use some colour to indicate the patient and the victim so that you could more easily identify them. There’s lots and lots and lots of distractors*. (Public contributor 2)

One public contributor, in reference to a genomic tree presented vertically and one presented horizontally, said:

*How it is spaced within the page is going to be of vital importance to make sure that you get the best chance of people understanding it*. (Public contributor 3)

Developing from this discussion, there was also a consideration of whether it would be better to draw the phylogenetic trees from the bottom or from the top, in the same way that family trees were presented:

Public contributor 1: *… I would definitely agree that the vertical is better. But should it spring from the bottom, or should it spring from the top? So I mean, maybe we’d need to do a random sample to ask people*.

Public contributor 4: *Maybe springing it from the bottom, would actually make people stop and think, and not jump to the conclusions that they do when you start from the top, which is how we see, you know Kings and Queens of England…*

Contributors highlighted the need for PPI across pathogen genomics, with key roles identified in research prioritisation, data practices and communication, forming the basis of the conclusions presented below.

## Conclusions

Although pathogen genomics research is complex, this project identified when, where, how and why PPI could be integrated into pathogen WGS. The findings demonstrate that, due to its complexity, collaborative sense-making and the development of shared language are essential early steps in meaningful public involvement. These processes should themselves be recognised as valuable forms of involvement, as they enhance researchers’ explanations and support the accessibility of complex concepts. Sense-making can be supported by using simple, clear and consistent terminology alongside appropriate visual aids. Although this project did not evaluate learning progression, future PPI work could build on previous research exploring how phylogenies are interpreted [24,27] to co-develop phylogenetic trees with public input, thereby improving the accessibility of pathogen genomics data visualisation.

Many of the areas of contribution identified by the public were not in the practicalities of how laboratory work is done for pathogen genomics, but in the targeted application of this technology. In many ways, the priorities for PPI in pathogen genomics identified in this study echo themes already identified in human genomics, where legal, ethical and social issues in genomics, as well as data governance, are often explored [28,29]. Pathogen genomics could learn from its close relative, as well as identifying other areas specific to PPI in pathogen genomics in which the public clearly has an interest, such as disease transmission or antibiotic resistance.

Recent outbreaks of infectious diseases such as COVID-19 and monkeypox have had high media profiles, increasing public exposure to pathogen genomics [30,31]. This, combined with the increasing use of pathogen WGS in diagnostic work and routine public health practice, creates an opportunity for PPI in future pathogen genomic studies [19]. Expanding PPI in pathogen genomics could help align pathogen WGS research with patient priorities, inform approaches to data governance and improve the accessibility of research outputs to the public. Researchers in pathogen genomics should embrace PPI in their work to reap the many benefits it could bring.

## Supplementary material

10.1099/mgen.0.001691 Uncited Supplementary Material 1.

## Abbreviations

DNA: deoxyribonucleic acid
HPRU: Health Protection Research Unit
NIHR: National Institute for Health and Care Research
PPI: patient and public involvement
UKHSA: UK Health Security Agency
WGS: whole-genome sequencing
