# Public and patient involvement: exploring public partnership in pathogen whole-genome sequencing research and its data visualisation

**DOI:** 10.1099/mgen.0.001763

**Published:** 2026-06-18

**Authors:** Suzanne Rotheram, Hannah Trivett, Jane Whitehurst, Caitlin Collins, Jake Carson, Sophie Staniszewska, Xavier Didelot

**Affiliations:** 1 Department of Public Health, Policy and Systems, The University of Liverpool, Liverpool, UK; 2 Department of Microbes, Infection & Microbiomes, School of Infection, Inflammation and Immunology, College of Medicine & Health, University of Birmingham, Birmingham, UK; 3 Public Contributor,, Warwick, UK; 4 UK Health Security Agency, London, UK; 5 Mathematics Institute, University of Warwick,, Coventry, UK; 6 Warwick Medical School, Division of Health Sciences, University of Warwick, Coventry, UK; 7 School of Life Sciences and Department of Statistics, University of Warwick, Coventry, UK

In the original version of this article author Sophie Staniszewska was incorrectly listed as Sophie Staniszeweska.

The author list has been corrected accordingly in both this correction notice and the Version of Record.

The authors apologise for any inconvenience caused.

