# Supplementary material for: Public and patient involvement: exploring public partnership in pathogen whole-genome sequencing research and its data visualisation
Source: Microb Genom. 2026 Apr 9;12(4):001691. doi: 10.1099/mgen.0.001691 (PMC13071077; doi:10.1099/mgen.0.001691)
Supplement: Uncited Supplementary Material 1. [file mgen-12-01691-s001.pdf]

Appendix A: Visual minutes from workshop 1

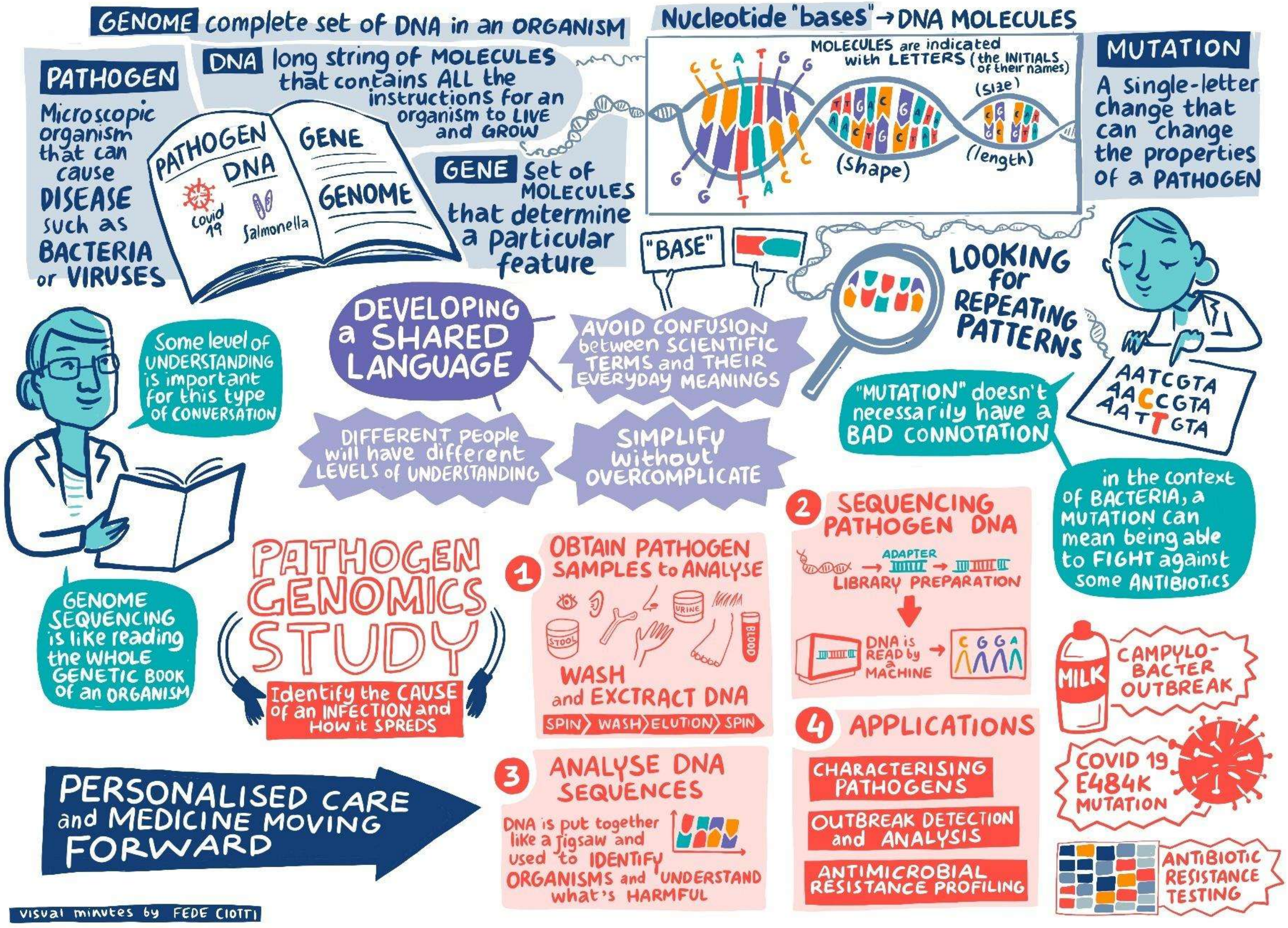

Appendix B: Visual minutes from workshop 2

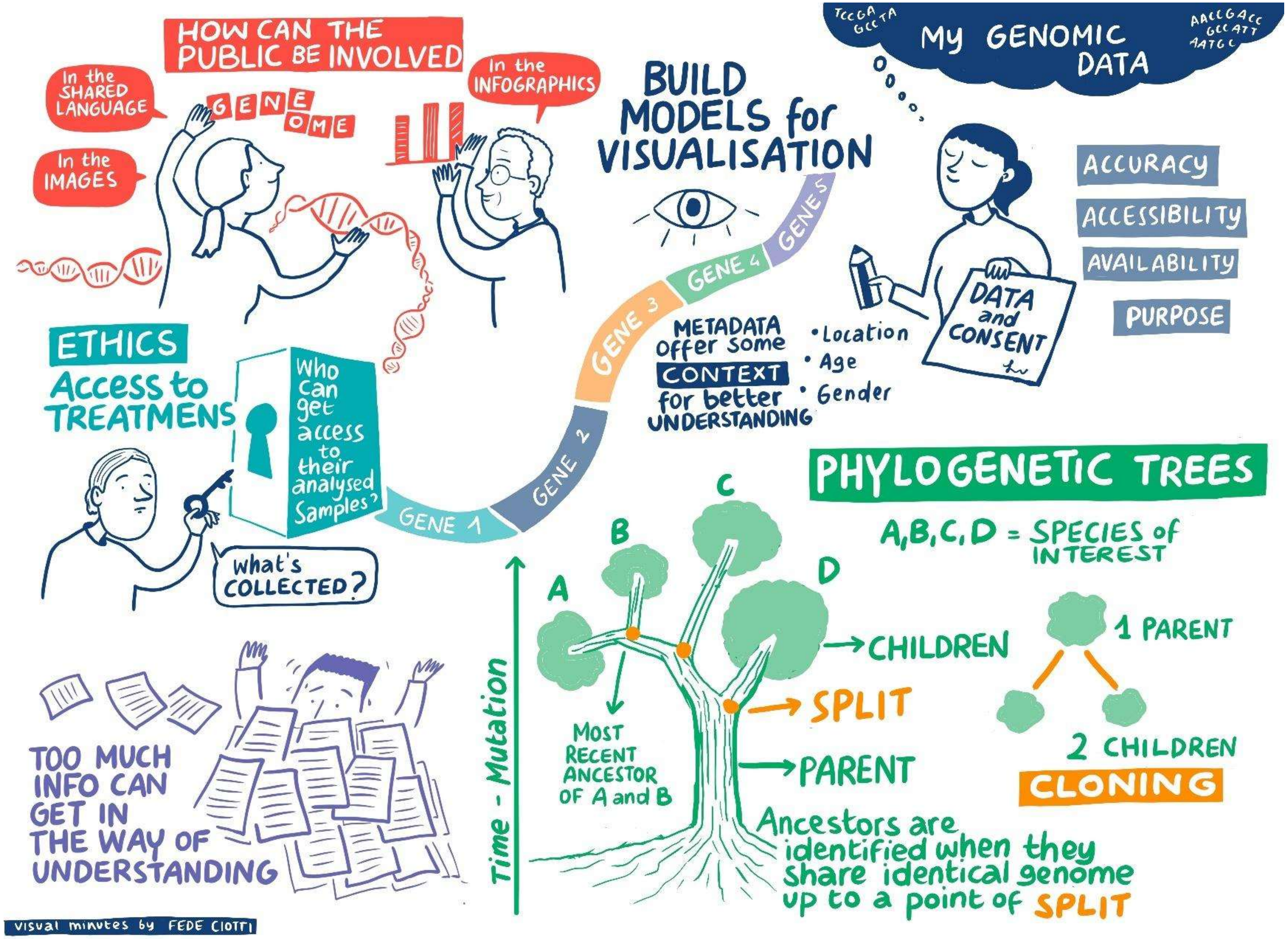

Appendix C: Visual minutes from workshop 3

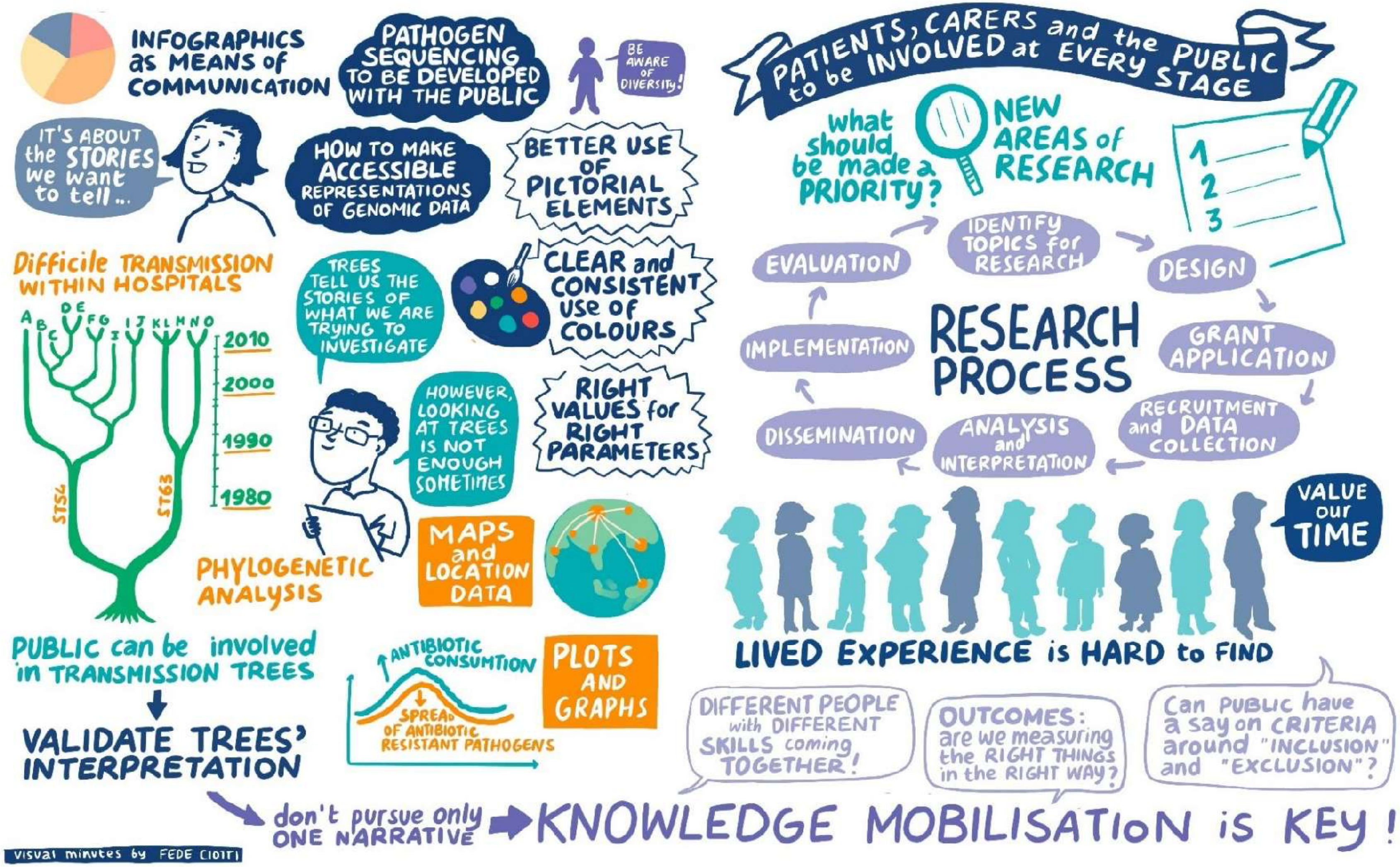

Appendix D: 'Living' Dictionary developed during sessions

Term/Word	Definition
**Analysing DNA**	Putting DNA sequences together like a jigsaw
**Antibiotic Resistance**	When bacteria are no longer sensitive to the drugs designed to kill them
**Antibiotic Resistance Profiling**	To establish what antibiotic resistance there is in bacteria
**Antimicrobial**	Drug that kills bacteria and other germs
**Antimicrobial Resistance (also known as AMR)**	When bacteria and other microbes (e.g. viruses) are no longer sensitive to the drugs designed to kill them
**Antimicrobial Resistance Profiling**	To establish what antimicrobial resistance there is in bacteria and other microbes
**Bacteria**	Type of germs, some keep you healthy, some make you sick
**Bacterial**	Having to do with bacteria
**Bacterial diversity**	Extent to which differences exist in a given set of bacteria
**Bacterial Species**	One type of bacteria with the same group name, a bit like a family of bacteria e.g. *Salmonella* species
**(Nucleotide) Base**	The basic building blocks of DNA (represented by the letters A, T, G or C)
**Broad Spectrum Antibiotic**	An antibiotic that can target many types of bacteria
Campylobacter	A bacteria which is a common cause of stomach bugs caused by food
**Colony**	A group of bacteria of the same species i.e. Salmonella species, when grown in the laboratory
**Commensal Organism**	Organisms that form beneficial partnerships with their human hosts (e.g. Bacteria in the gut or on the skin)
**Common Ancestor**	An ancestor that is shared by a group of organisms
**DNA**	The genetic code at the centre of cells which instructs their microscopic machinery to make all the proteins that make them. A long string of molecules on a double helix shape made up of repeating combinations of molecules – represented by the letters A, T, G & C
**DNA Preparation: Wash**	Breaking apart the bacteria, detaching the unwanted material to make the bacteria DNA clean
**DNA Preparation: Elution**	The clean DNA is transferred into a clean liquid with no extra materials so the DNA is pure and ready to use
**Encoding**	Relationship between a gene and the protein it allows microbes to make
**Evolutionary Trees**	Representation of the ancestral relationships between a set of organisms
**Genealogy**	Tracing the pathogen ancestry back in one line from an initial sample
**Gene**	A short stretch of DNA that provides the instruction for the production of a specific protein
**Germ**	A microorganism which makes you sick
**Genetic Code**	The DNA code that is inherited in their genes
**Genetics**	Having to do with genes

**(Whole) Genome Sequencing**	The process by which scientists read an organism's genetic code
**Genome**	The entire genetic code of an organism
**Genomic Data**	A collection of genetic sequences
**Genomic Surveillance**	Using genomic data to monitor infectious diseases
**Metadata**	Extra information collected alongside the genomic or sequencing data. E.g. age, gender, address, post-code etc.
**Metagenomic Sequencing**	To take a snapshot of multiple microbes DNA in the sample simultaneously using a sequencing machine
**Microbe**	A very small organism
**Mutation**	A permanent change in the DNA sequence of a gene, leading to an alteration in the protein encoded by the gene
**Node**	The point at which an evolutionary tree branches
**Notifiable infection**	An infection which must be reported because it could cause a severe public health problem
**Organism Evolution**	Genetic changes that happen over time
**Organism**	A living entity
**Origin of Replication**	The start of the gene sequence
**Outbreak**	Unusually high number of cases of an infectious disease that are linked by a common cause
**Pathogen**	Any microscopic organism that can make you sick
**Pathogenicity**	The ability of a microbe to cause disease
**Pathogenic Organism**	Same as pathogen
**Patient Zero**	The first person to have a particular infection
**Petri dish**	Container used to grow bacteria in laboratories
**Phylogenetic Trees**	Same as evolutionary trees
**Plasmid**	Small part of circular DNA found in a bacteria
**Plates**	e.g. agar plates - used to grow bacteria
Salmonella	A family of bacteria which can cause disease
**Sanger Machine**	Specific machine that can be used to read the DNA of an organism
**Sequencing Machine**	Any machine that can be used to read the DNA of an organism
**Virulence**	The ability of any infective agent, or pathogen, to cause a disease, or a measure of the severity of the disease that the pathogen causes
**Virus**	A piece of genetic code that can reproduce itself and therefore multiply and spread

**Words used interchangeably**
pathogen = germ
Base = nucleotide
Whole genome sequencing = genome sequencing = sequencing
Phylogenetic tree = evolutionary tree = genetic tree = phylogeny

Appendix E: The NIHR PPI research cycle, source (22)

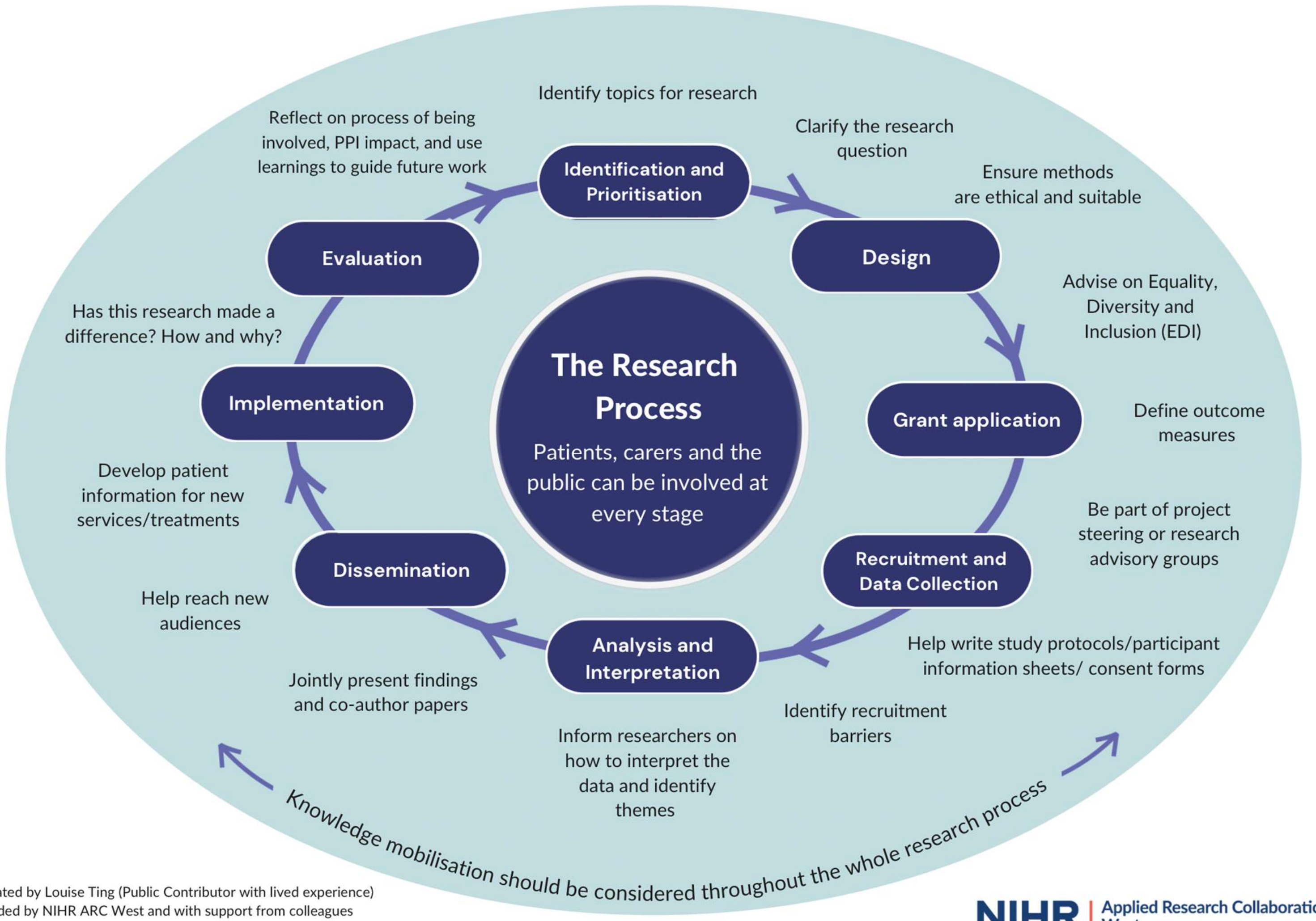

Created by Louise Ting (Public Contributor with lived experience)
Funded by NIHR ARC West and with support from colleagues
https://arc-w.nihr.ac.uk/working-effectively-with-public-contributors/

NIHR | Applied Research Collaboration West
